# Domestic and Community Violence in Greece After the First COVID-19 Quarantine: A Clinical Forensic Approach

**DOI:** 10.7759/cureus.46054

**Published:** 2023-09-27

**Authors:** Konstantinos Katsos, Christoforos Kolentinis, Ioanna Anastopoulou, Dimitrios G Vlachodimitropoulos, Nikolaos D Goutas, Chara A Spiliopoulou, Emmanouil I Sakelliadis

**Affiliations:** 1 Department of Forensic Medicine and Toxicology, National and Kapodistrian University of Athens School of Medicine, Athens, GRC

**Keywords:** clinical forensic medicine, injuries, forensic clinical examination, community violence, domestic violence, quarantine, covid-19

## Abstract

Many scientists expressed their concerns regarding the impact of COVID-19-related quarantine measures on interpersonal violence, mainly concerning children and intimate partners, as well as other negative psychological effects. During early 2020, free circulation in Greece was prohibited for 42 days, up until May 4th.

The aim of our study was to investigate characteristics of bodily harm allegation cases referred to the Department of Forensic Medicine and Toxicology of the Medical School of the National and Kapodistrian University of Athens, Greece, during the first month succeeding free circulation re-establishment in the broader Attica region. We also aimed to detect any possible differences regarding bodily harm allegations by comparing the corresponding time period of 2019.

A decrease in community violence (CV) allegations, especially youth violence incidents, was observed in 2020. Females' victimization, as well as allegations against strangers, were also decreased. No differences were observed concerning the injury mechanism. Victims of 2020 filed the allegations faster and, thus, were examined almost one day earlier than their 2019 counterparts.

During lockdown, domestic violence (DV) hotline reporting was significantly increased, but paradoxically DV cases referred to our Department were decreased. In Greece, the legislators did not foresee any specific exemption from circulation restriction for DV victims attempting to escape abuse. Our results revealed a small, but notable, impact on non-fatal interpersonal violence.

## Introduction

The first case of SARS-CoV-2 infection in Greece was diagnosed on February 26th, 2020. The Greek Government implemented a rather aggressive stance, to reduce COVID-19 spread, with the gradual closing of public and private sectors, which finally led to a general lockdown and restriction of free circulation, on March 23rd, 2020 [1]. During the period of restrictive measures, people were able to move outside their homes only for very specific reasons, such as to receive medical treatment, to purchase supplies (medications, food supplies, etc.), to perform bank transactions (when not feasible electronically), to provide assistance to persons in need, to attend ceremonies (e.g., weddings, funerals, baptisms but in all cases with very limited attendees), to visit children (in case of separated or divorced parents), to perform physical exercise (near the residence), and finally to perform companion animal walking. Before exiting their houses, they were obliged to send an SMS or to complete a specific "movement form" [2]. Free circulation was prohibited for 42 days, up until May 4th, 2020.

Many scientists expressed their concerns regarding the impact of restriction and quarantine measures on interpersonal violence, especially against children or intimate partners (intimate partner violence (IPV)) [3-11]. Furthermore, social distancing and prolonged stay-at-home orders were thought to lead to negative psychological effects (e.g., anxiety, anger, depression, substance use, fear, suicidality, and sleeping disorders) [12-15]. A similar negative psychological impact was previously encountered in the 2013-2016 Ebola outbreak in Africa [16] and was just recently confirmed by a Chinese study performed after the first COVID-19 outbreak [17].

Greece is a European country with approximately 10,000,000 inhabitants. Studies concerning interpersonal violence, and especially IPV and child abuse, are, therefore, extremely scarce. IPV allegations in our Department's jurisdiction are rare (one allegation per 10,000 habitants every year), while allegations of violence against minors represent less than 10% of all cases [18,19].

Clinical forensic examinations in our Department are performed within the context of preliminary judicial investigation, only after an allegation of domestic violence (DV) or community violence (CV), either has been submitted either to the Public Prosecutor or the Police. DV incidents include cases subjected to the Greek Law about DV (intimate partners, children, parents, and siblings), while CV incidents include interpersonal violence cases other than DV subjected to the Greek Penal Code. Youth violence includes CV cases in which at least one person involved belongs to the 10-29 years age group.

The aim of these examinations is injury recording and assessment. Our Department’s jurisdiction area covers approximately one-tenth of the entire Greek population. Typically, more than 500 clinical forensic examinations are performed annually (the mean monthly number of forensic examinations performed is approximately 40).

According to the Greek General Secretariat for Family Policy and Gender Equality, during the lockdown, hotline calls for DV almost quadrupled in April 2020 compared to March 2020 [20], while according to our Department data, a paradoxical 56% decrease in clinical forensic examinations related to bodily harm allegations for interpersonal violence was observed in the same period, when compared to 2019 (55 cases in 2019 vs. 24 in 2020).

In this article, we aim to investigate the characteristics and any possible differences in interpersonal violence-related bodily harm allegations during the first month (from May 4th to May 31st, 2020) after free circulation re-establishment in the Attica metropolitan region, in comparison with the corresponding 2019 time period.

## Materials and methods

For this retrospective study, we included clinical forensic examination cases performed in our Department, concerning incidents from May 4th to May 31st, 2019, and from May 4th to May 31st, 2020. For every bodily harm allegation, we recorded the following variables: victim’s and perpetrator’s demographic characteristics (sex, age, nationality, employment status); incident day (working day, holiday); victim’s attendance at an ED for primary care, prior to the forensic examination; body region affected; time period (in days) between the incident and the allegation and between the incident and the forensic examination; injury mechanism (a: physical violence, when just a body part acted as a blunt force instrument, b: use of a blunt force object only, c: a combination of a and b, d: sharp force instrument, e: sexual abuse, and f: firearm); and injury characterization according to the Greek Penal Code (a: grievous injuries that caused the victim a life-threatening or severe or long-term illness or serious mutilation or injuries that prohibited the use of the victim’s body or mind for a long time period, b: dangerous injuries that were inflicted in a manner that could cause the victim severe injuries, and c: actual injuries that included injuries not considered to be severe or dangerous). Furthermore, cases were divided into two groups: DV incidents and CV incidents. The study was approved by the Ethics Committee of the Medical School of National and Kapodistrian University of Athens, Greece (approval number 334).

Data was collected anonymously and is presented as absolute and relative (%) frequencies. Due to the sample size, Fisher’s exact test was chosen for the statistical comparison of the categorical data obtained. Whenever a variable was unknown for a case, this case was excluded from the specific statistical analysis. Data analysis was performed using SPSS Statistics version 25.0 (IBM Corp. Released 2017. IBM SPSS Statistics for Windows, Version 25.0. Armonk, NY: IBM Corp.). A value of p<0.05 was considered statistically significant.

## Results

From May 4th to May 31st, 2020, a total of 29 bodily harm allegations were referred to our Department. Comparing the cases of the two examined periods, a CV decrease (p=0.6067), especially concerning youth violence incidents (p=0.0145), was observed in 2020, while DV remained unaffected (Table 1).

**Table 1:** Types of bodily harm allegations *Allegations for youth violence include CV cases in which at least one person involved belongs to the 10-29 years age group CV: community violence, DV: domestic violence

	2019 (n=37)	2020 (n=29)	Fisher exact test p-value
Allegations of CV	25 (67.6%)	17 (58.6%)	0.6067
Allegations of youth violence*	16 (43.2%)	4 (13.8%)	0.0145
Allegations of DV	12 (32.4%)	12 (41.4%)	0.6067
Allegations of intimate partner violence	8 (21.6%)	7 (24.1%)	1
Allegations of child abuse by a parent	2 (5.4%)	3 (10.3%)	0.647
Allegations of parental abuse by a child	2 (5.4%)	0	0.4998
Allegations of abuse by a sibling	0	2 (6.9%)	0.1893

Furthermore, allegations of females' victimization (p=0.1361) and allegations against strangers (p=0.4436) were decreased (Table 2).

**Table 2:** Victims and perpetrators' demographic characteristics and other variables of the interpersonal violent incident * Statistical analysis for employment status, both for victims and perpetrators, was performed by grouping the unemployed, the retired, and the students in one category and comparing this group with the employed ** The sole incident of victimization by a male and a female in 2019 was not included in the statistical analysis for the perpetrator's sex *** The incidents with unknown values, such as the perpetrator's nationality and perpetrator's employment status were not included in the statistical analysis

		2019 (n=37)	2020 (n=29)	Fisher exact test p-value
Victim's sex	Male	15 (40.5%)	18 (62.1%)	0.1361
	Female	22 (59.5%)	11 (37.9%)	
Victim's age	<17 y	5 (13.5%)	5 (17.2%)	0.7383
	≥18 y	32 (86.5%)	24 (82.8%)	
Victim's nationality	Greek	35 (94.6%)	28 (96.5%)	1.0000
	Other	2 (5.4%)	1 (3.5%)	
Victim's employment status	Employed	20 (54.1%)	17 (58.6%)	0.8046*
	Unemployed	3 (8.1%)	2 (6.9%)	
	Retired	3 (8.1%)	5 (17.2%)	
	Student	11 (29.7%)	5 (17.2%)	
Perpetrator's sex	Male	31 (83.8%)	23 (79.3%)	0.5201
	Female	5 (13.5%)	6 (20.7%)	
	Male and female	1 (2.7%)	0	**
Perpetrator's age	<17 y	3 (8.1%)	2 (6.9%)	1.0000
	≥18 y	34 (91.9%)	27 (93.1%)	
Perpetrator's nationality	Greek	32 (86.5%)	22 (75.9%)	0.0680
	Other	2 (5.4%)	7 (24.1%)	
	Unknown	5 (13.5%)	0	***
Perpetrator's employment status	Employed	16 (43.2%)	15 (51.7%)	1*
	Unemployed	3 (8.1%)	4 (13.8%)	
	Retired	2 (5.4%)	2 (6.9%)	
	Student	4 (10.8%)	2 (6.9%)	
	Unknown	12 (32.4%)	6 (20.7%)	***
Perpetrator's familiarity with the victim	Known	21 (56.8%)	20 (69.0%)	0.4436
	Stranger	16 (43.2%)	9 (31.0%)	
Day of the violent incident	Working day	31 (83.8%)	19 (65.5%)	0.1466
	Holiday	6 (16.2%)	10 (34.5%)	
Attendance at the ED		11 (29.7%)	15 (51.7%)	0.0815

During the 2020 study period, we noted two allegations of police misconduct not recorded during 2019. Furthermore, two cases related to non-compliance with personal protective measures within public transportation were recorded in 2020, both of which represent a novel CV etiology. More specifically, in the first case, a Police Officer was attacked by a passenger not wearing a mask, and another passenger was injured by a public transportation employee for the same reason. Both incidents took place in the subway, on the same day, but at different stations and hours.

No differences were observed concerning the injury mechanism, as in most incidents, perpetrators have used physical violence (either alone or in combination with a blunt force instrument), for both study periods (89.2% in 2019 and 86.2% in 2020). No firearm injuries were recorded for either study period. Three sexual assault allegations were recorded in total (one in 2019 and two in 2020), as well as two allegations of human bites (one per year).

Victims in 2020 filed the allegations earlier (the mean period between incident and allegation submission was 1.41 days for 2019 and 0.45 days for 2020) (p=0.0725) and, thus, were examined almost one day earlier than those of 2019 (the mean period between incident and forensic examination was 2.35 days for 2019 and 1.65 days for 2020) (p=0.8074).

As demonstrated in Table 3, a decrease in head (p=0.3325), torso (p=0.6181), and lower limb (p=0.0815) injuries was observed.

**Table 3:** Anatomical distribution of injuries and their legal characterization

	2019 (n=37)	2020 (n=29)	Fisher exact test p-value
Anatomical distribution of injuries			
No injuries	1 (2.7%)	3 (10.3%)	0.3122
Head	20 (54.1%)	12 (41.4%)	0.3325
Neck	4 (10.8%)	5 (17.2%)	0.4908
Torso	17 (45.9%)	11 (37.9%)	0.6181
Upper limbs	17 (45.9%)	20 (69.0%)	0.0820
Lower limbs	26 (70.3%)	14 (48.3%)	0.0815
Legal characterization of injuries			
Simple (actual) injuries	31 (83.8%)	20 (69.0%)	0.2365
Dangerous and severe (grievous) injuries	5 (13.5%)	6 (20.7%)	0.5153

## Discussion

To the best of our knowledge, despite the existing COVID-19 literature and its multiple negative effects, no studies have yet been published concerning the impact of restriction and quarantine measures on interpersonal violence after the lockdown ended. These novel circumstances were thought to have a negative psychological impact and consequently affect interpersonal violence incidents.

Despite these expressed concerns, our study demonstrated some paradoxical results. The most notable was the decrease in CV allegations. The latter may imply that people were less willing to quarrel, especially with a stranger. The increased negative psychological effects of the 42-day quarantine, including fear of contagion and death, may have acted as a catalyst for aggression expression toward others [21] after the lockdown ended but only for the first post-lockdown days. On the other hand, the decrease observed in youth violence allegations may suggest a less negative psychological impact within this specific age group (10-29 years old). In any case, this result is yet to be confirmed by more studies. Individuals belonging to this age group were probably relieved when the lockdown ended, as they could finally interact in person with their friends and relatives.

After the lockdown ended, all temporarily closed enterprises gradually re-opened, with coffee shops, bars, and restaurants re-opening at the end of May 2020. Taking into consideration that during weekends and holidays, Greeks are used to having lunch in restaurants and spending leisure time in coffee shops and bars, the observed increase in quarrels during these days may be, partially, explained because of increased stress and frustration due to the unprecedented circumstances.

Another paradoxical finding of our study was the increased ED attendance after the incident and prior to the forensic examination, despite expressed thoughts of unwillingness to visit hospitals, which are thought to be placed with a high risk of infection [8,22], possibly due to general public perception that the pandemic in Greece was handled, at the time, more than adequately.

During the period of our study, no non-fatal gun violence was observed. According to the results of a recent study from our Department, non-fatal firearm injuries are extremely rare (1.2%) in the clinical forensic context, probably due to the strict regulations concerning gun ownership in Greece. On the contrary, US researchers reported a paradoxical increase in gun violence during the COVID-19 pandemic [23,24].

The findings concerning DV cases referred to our Department demonstrated the most paradoxical results. During the lockdown (the period prior to our study period), a significant decrease in all interpersonal violence-related allegations was observed, while during our study period (the first month after the lockdown ended), DV cases remained unaffected. Concerning the lockdown period, it is plausible that the absence of specific guidance for DV victims attempting to escape abuse has accounted for the inconsistency between the decreased cases examined in our Department and the observed hotline traffic increase. According to Mahase [25], such guidance was issued during the lockdown in the UK. Another possible explanation is that the increased hotline traffic during the lockdown was related more to psychological violence than to any serious injuries. It should also be noted that during the lockdown, Courts were open only for urgent cases, and people could visit a police station only after arranging an appointment. One would assume that as soon as free circulation was re-established (the time period of our study), most DV victims would seek both medical and legal help. Therefore, an increase in DV cases referred for clinical forensic examination was to be expected.

In our study, most IPV victims were women (eight cases in 2019 and five cases in 2020). In general, but also possibly in our study sample, cultural and religious beliefs, fear of revenge, lack of economic and social support, stigmatization, fear of losing children custody, and hope that the partner will change in the future may provide an explanation as to why women do not abandon their violent partners [10,26] and consequently do not file an allegation against their abusers. According to our experience, many women find the courage required to file an allegation against their abuser and to submit to a forensic clinical examination only after several violent incidents have occurred in the past. Injuries inflicted in these past incidents are typically more severe than the current, under-examined ones. Dissatisfaction and frustration with the justice system, as well as delays in prosecutions, may also partly prove to be a reason why victims in the broader Attica region were still underreporting their victimization to the authorities.

Since 2006, and the implementation of Greek Law about DV, bodily harm in the domestic context has been considered an ex officio prosecuted crime. Thus, police officers are legally obligated to arrest a DV perpetrator, even if the victim does not file an allegation [27]. Therefore, police officers responding to a DV incident should receive adequate training to appropriately handle such cases.

The hypothesis that increased hotline traffic should have led to increased DV allegations was not confirmed by our study, suggesting that current theories and policies should be reconsidered. Even though DV, especially IPV, represents a global phenomenon, specific policies for each population should be adopted, based on their special needs [19]. We believe that similar studies should be conducted in several countries to clarify the existence of similar phenomena. Furthermore, the declining trend of engagement in quarrels with strangers in CV incidents should also be examined as it may represent an actual change due to the novel circumstances.

According to our results, lockdown had a small but notable impact on non-fatal interpersonal violence. As people are nowadays more informed, any possible lockdown should be more easily accepted. Official guidance for DV victims should be issued prior to the enactment of any new restriction measures, as was the case in other European countries. Clinicians' awareness of interpersonal violence victims during epidemics should be raised enough to allow victim identification and adequate response (especially for DV victims and children, adolescents, and young adults) [28,29]. Trained psychiatrists, psychologists, and workers in social and legal departments, as well as other experts, should combine their efforts to prevent or at least manage the DV crisis [21]. Police officers should be adequately re-informed and re-trained to deal with this different and novel criminal phenomenon [30].

## Conclusions

The reported increased hotline traffic was not confirmed by our study and did not record an increase in DV allegations filed. This calls for a reconsideration of current theories and policies. Future planning should take into consideration all aspects of the subject in hand to ensure improved awareness of all relevant personnel, including medical and legal professionals, as well as police officers.
